# Ingestion and excretion dynamics of microplastics by black soldier fly larvae and correlation with mouth opening size

**DOI:** 10.1038/s41598-023-31176-9

**Published:** 2023-03-16

**Authors:** Siebe Lievens, Evelynn Vervoort, Daniele Bruno, Tom Van der Donck, Gianluca Tettamanti, Jin Won Seo, Giulia Poma, Adrian Covaci, Jeroen De Smet, Mik Van Der Borght

**Affiliations:** 1 Department of Microbial and Molecular Systems, Faculty of Engineering Technology, Research Group for Insect Production and Processing, KU Leuven – Campus Geel, Kleinhoefstraaat 4, 2440 Geel, Belgium; 2 Department of Pharmaceutical Sciences, Faculty of Pharmaceutical, Biomedical and Veterinary Sciences, Toxicological Centre, University of Antwerp – Campus Drie Eiken, Universiteitsplein 1, 2610 Wilrijk, Belgium; 3 Department of Biotechnology and Life Sciences, University of Insubria, 21100 Varese, Italy; 4 Department of Materials Engineering, Faculty of Engineering Science, KU Leuven – Campus Arenberg, 3001 Leuven, Belgium; 5 Interuniversity Center for Studies On Bioinspired Agro-Environmental Technology (BAT Center), University of Napoli Federico II, 80055 Portici, Italy

**Keywords:** Biological techniques, Environmental sciences

## Abstract

Black soldier fly (BSF) larvae (*Hermetia illucens*) are voracious feeders that can be reared on food waste streams originating from the food industry and retailers. Because these food waste streams are automatically being unpacked in substantial amounts, they can contain microplastics, potentially jeopardising the larvae’s chemical safety when applied as compound feed ingredients. During this study, the dynamics of ingestion and excretion of microplastics by BSF larvae reared on substrates containing different contents (*w*_MP_ = 0.00, 0.01, 0.10, 0.50, 1.00, 3.00%) of fluorescent blue-labelled microplastics (median size, *Dv(50)* = 61.5 µm) were monitored. To correlate the particle size with their uptake, larval mouth opening dimensions were measured during the rearing process. In conclusion, it appeared that ingestion of microplastics by BSF larvae depends on initial particle load, mouth size, and consequently also age. The larvae took up between 131 (*w*_MP_ = 0.01%) and 4866 (*w*_MP_ = 3.00%) particles leading to bioaccumulation factors (BAF) between 0.12 (*w*_MP_ = 3.00%) and 1.07 (*w*_MP_ = 0.01%). Larvae also appeared to excrete the microplastics, lowering the BAFs to values between 0.01 (*w*_MP_ = 3.00%) and 0.54 (*w*_MP_ = 0.01%).

## Introduction

*Hermetia illucens,* also known as the black soldier fly (BSF), is an insect species with a holometabolous life cycle consisting of four life stages. The life cycle of the BSF spans, depending on the rearing substrate, 40–45 days^1–3^. Although the adult fly is endowed with well-developed mouthparts and a functional alimentary canal, they do not consume food^4^. In contrast, the larvae of the black soldier fly are voracious feeders. This means they can be reared on low- to high-quality waste streams, ranging from manure to retailer waste^5^, converting these streams into valuable biomass^6^. They also have a high fat and protein content, and contain essential amino acids, vitamins and minerals, which gives them great potential as a feed compound ingredient^7,8^. Given the potential of the BSF larvae to grow on several residual streams, there is also an increasing concern about the microplastics which are potentially present in these types of streams. Food waste originating from, for example, the food industry (e.g. rejected or spoiled products, etc.) and leftovers from supermarket and catering services are often wrapped in plastic packaging materials. These products are collected by organic waste management companies who use de-packaging equipment to separate the food waste from the packaging materials^9^. Those machines have capacities ranging from 5 to 20 tons/day^10^, allowing significant quantities of food waste to be processed. The unpackaging activity results in an organic slurry that can contain small amounts of plastics of various sizes. Due to its high organic content, this slurry is suitable for the production of biogas, while its residue can be utilised as organic fertiliser^9,11^. Besides the use of food waste streams as biogas precursors, food waste can also be applied as rearing substrates for BSF larvae, which can in turn be used as a valuable ingredient in compound feed (preparing for re-use). The latter corresponds to a higher value product compared to energy generation according to the waste hierarchy management presented by the EU Directive 2008/98/EC^12^. However, an essential requirement of using food waste to produce BSF larvae is that the presence of plastic particles in the insect feeding substrate does not compromise the safety of insects as feed ingredients.

In the study of Lievens et al.^13^, BSF larvae were exposed to various concentrations of polyvinyl chloride (PVC) macro-, meso-, and microplastics. In general, the presence of PVC in the substrate did not affect the larval growth. From these results, new questions arose, including whether BSF larvae are capable of ingesting and/or excreting such microplastics. Therefore, this study aims to understand the dynamics of microplastic ingestion and excretion by BSF larvae, which is currently lacking. Specifically, a method was developed to visualise and quantify fluorescent blue-labelled polyethylene microplastics in the lumen of the alimentary canal of BSF larvae. Subsequently, BSF larvae were reared on an artificial food waste (a mixture of fruits, vegetables, dairy products, and bread)^9^ spiked with different contents of microplastics and the visualisation method was applied to quantify the ingestion and excretion of the microplastics. Furthermore, we hypothesised that a correlation can be made between the microplastic uptake, the mouth opening and age of BSF larvae. Therefore, the mouth opening size of BSF larvae of different ages was measured by scanning electron microscopy (SEM).

## Results and discussion

### Microplastic characterisation

**Figure 1:**
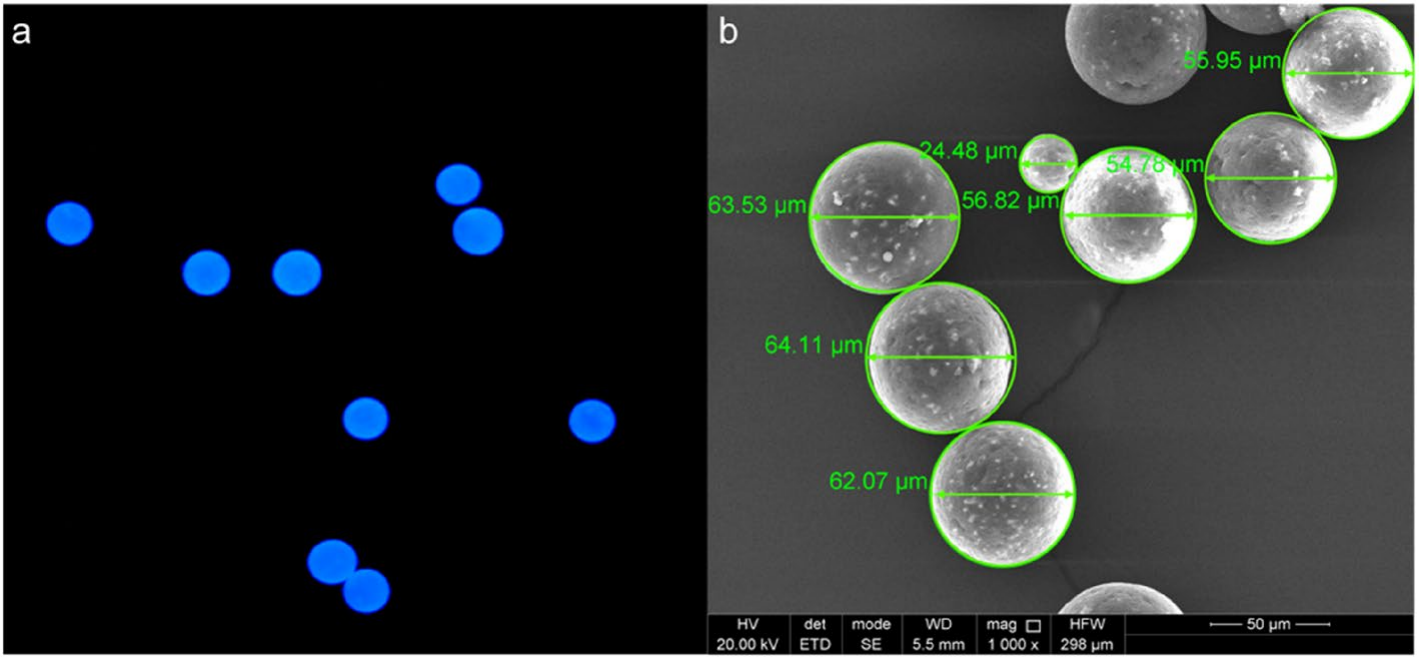
The fluorescent blue-labelled polyethylene microplastics visualised using (**a**) fluorescence microscopy (magnification: 10 x) and (**b**) scanning electron microscopy (magnification: 1000 x).

The blue-labelled polyethylene particles used in this study were distinctly spherical, with a smooth surface (Fig. 1) and a relatively small particle size distribution. Volume-weighted percentile values *Dv(10)*, *Dv(50)*, and *Dv(90)* were 46.6 ± 0.1 μm, 61.5 ± 0.1 μm, and 82.0 ± 0.4 μm, respectively, meaning that 10, 50 and 90% of the particles are smaller than the respective size. The median size of these blue-labelled particles (*Dv(50)* = 61.5 μm) corresponded with the particle size (53–63 µm) provided by the vendor, and was used for further bioaccumulation factor (*BAF*) calculations.

### Visualisation of microplastics in the gut of BSF larvae

A first step toward the assessment of microplastic ingestion and excretion by BSF larvae was the development of a detection method for microplastics in the lumen of the larval gut. In (semi-)transparent organisms like *Daphnia* spp., Pacific oyster larvae (*Crassostrea gigas*), and zooplankton, microplastics can be easily visualised^14–17^. Often digestion with nitric acid or hydrogen peroxide is required to remove biological matter prior to the quantification of the microplastics^14,15^. However, it is not possible with the latter method to differentiate between microplastics present in the gut and in other tissues or from the organism’s surface. Hence, since BSF larvae are not transparent, and in order to solely quantify the plastic particles present in the BSF larval gut, the gut must therefore be isolated prior to visualisation of microplastics.

**Figure 2:**
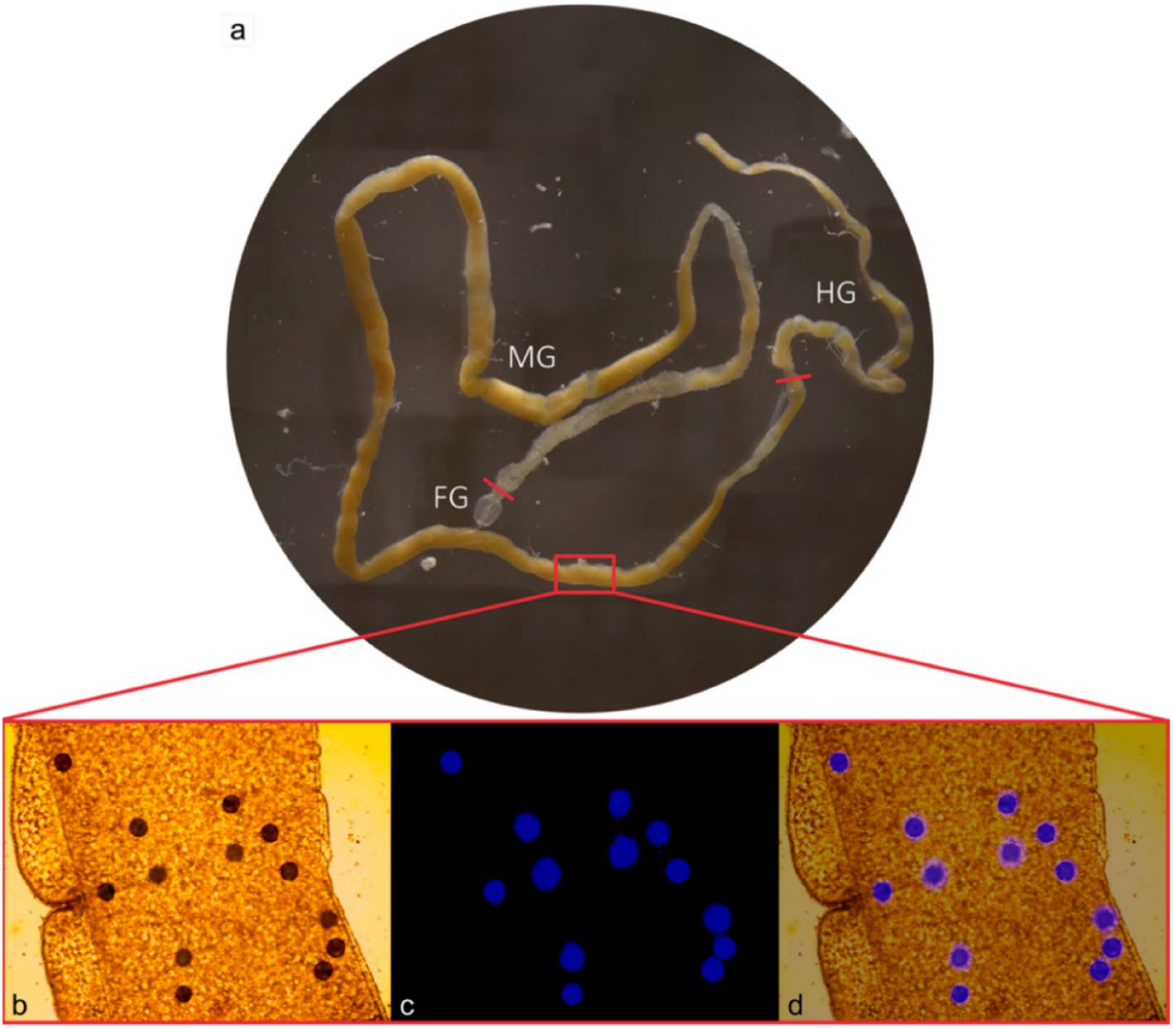
(**a**) Image of the complete gut and its different sections of a BSF larva as shown under a stereo microscope (magnification: 8 x). (**b**), (**c**), and (**d**) show different images of the same larval gut section (magnification: 10 x). (**b**) Light microscope image, (**c**) fluorescence microscope image, and (**d**) an overlay of both images, to visualise the microplastics present in the lumen of the larval gut. FG, MG, and HG represent the foregut, midgut, and hindgut, respectively.

BSF larvae were reared on artificial food waste containing blue-labelled polyethylene microplastics. During the rearing phase, a single larva was taken out of the rearing substrate and dissected. Next, the isolated gut, consisting of the different gut sections (foregut, midgut, and hindgut), was examined under a stereo microscope (Fig. 2a). Blue spots were clearly observed in the lumen of the gut. To verify that these blue spots were indeed the added fluorescent microplastics, gut sections were studied through an optical microscope (Fig. 2b), whereafter the same sections were examined using the fluorescent filter, only visualising the fluorescent microplastics (Fig. 2c). An overlay (Fig. 2d) of both images (Fig. 2b and c) revealed that the delineated round dark spots indeed corresponded to the fluorescent blue-labelled microplastics, enabling the quantification of microplastics in BSF larval guts using a light microscope. Further, the particles consistently held their spherical shape in the larval gut during the whole experiment. Therefore, it is hypothesised that no degradation took place, though this should be investigated in a follow-up study. Similar results were also obtained for another insect species i.e. Bombyx mori. In the latter study, different transversal cryosections were used to visualise the microplastics through microscope analysis, unfortunately thereby making quantification impossible^18^.

### Quantification of the microplastic ingestion by BSF larvae

**Figure 3:**
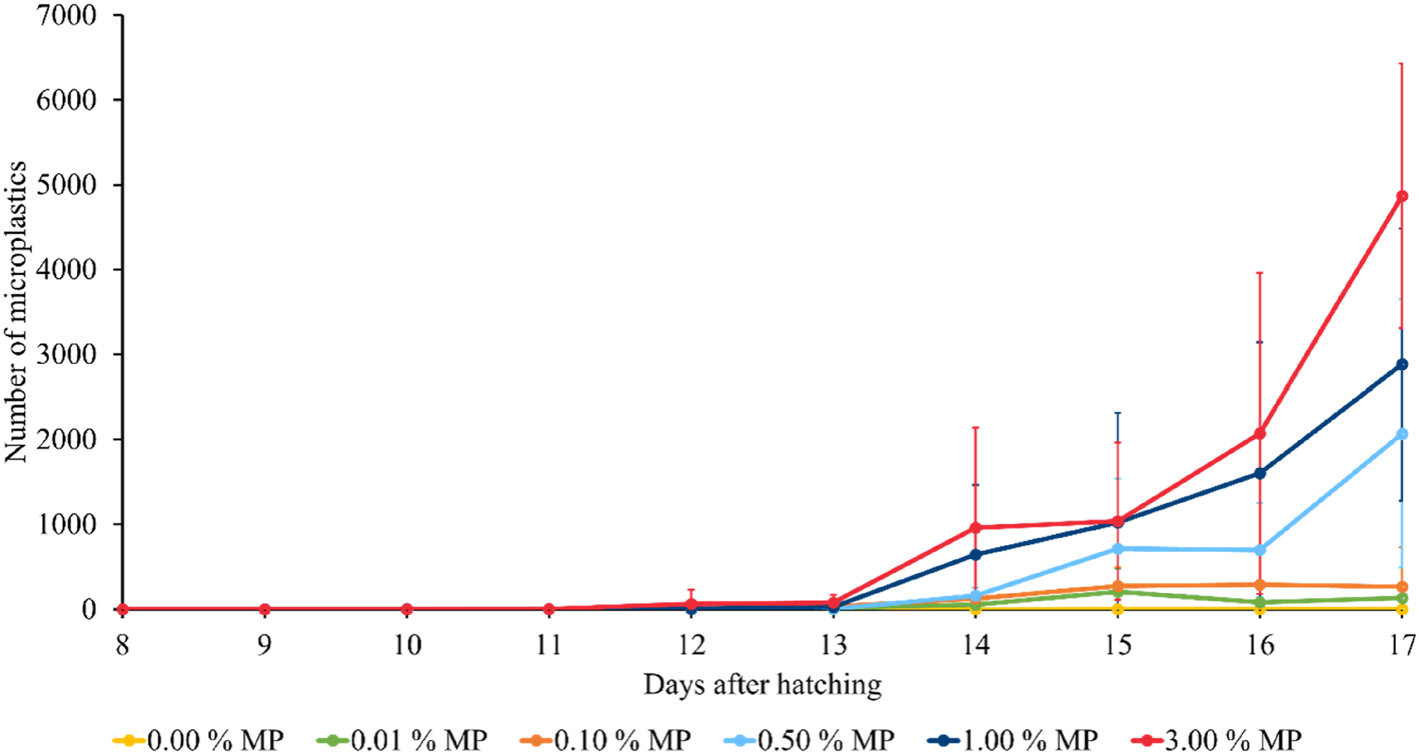
Uptake of microplastics (MP) over time by BSF larvae reared on an artificial food waste stream containing different microplastic contents (*w*_MP_ = 0.00, 0.01, 0.10, 0.50, 1.00, 3.00%) of fluorescent blue-labelled polyethylene microplastics (*Dv(50)* = 61.5 µm). The results shown are the mean values (*n* = 9), while the error bars depict their standard deviations.

After the visualisation and the confirmation of microplastics present in the gut of the BSF larvae, the ingestion of particles was quantified. For this purpose, larvae which were hatched 8 days earlier were used, further in the text referred to as 8 days after hatching or 8 DAH. These larvae were reared on a simulated food waste spiked with different contents (*w*_MP_ = 0.00, 0.01, 0.10, 0.50, 1.00, and 3.00%) of the above-mentioned fluorescent blue-labelled polyethylene microplastics (MP). During the rearing experiments, three larvae were taken out of each container daily. After isolating their gut, the number of plastic particles present in the gut lumen was counted under a light microscope as described above. Figure 3 shows the uptake of microplastics by BSF larvae. A similar pattern was observed for all substrate groups, except for the 0.01% and 0.10% microplastic groups on the last day of the experiment (17 DAH). In all cases, the ingestion of the spherical particles began slowly, at 10 DAH, and increased considerably starting from 13 DAH. The BSF larvae ended up with 4866 ± 1560 particles in their gut at the highest initial microplastic content (*w*_MP_ = 3.00%), whereas 2883 ± 1602 (*w*_MP_ = 1.00%), 2067 ± 1578 (*w*_MP_ = 0.50%), 264 ± 462 (*w*_MP_ = 0.10%), and 131 ± 187 (*w*_MP_ = 0.01%) particles were found for the other contents, showing that the final particle amount correlates with the initial microplastic content in the substrate. A similar, considerable increase was also seen for other species (e.g. *Daphnia* spp.), just as the final particle amount in the organism also depends on the initial microplastic content in their substrate^14,19^.

In the case of the two lowest microplastic contents (*w*_MP_ = 0.01% and 0.10%), a plateau was reached, with no further accumulation of microplastics after 15 DAH. A possible explanation is that the experiment involving the two lowest particle contents was conducted at another (later) date, which is further explained in the materials and methods section. Even though the rearing conditions (temperature, relative humidity, substrate, etc.) were the same, the larvae could have developed slightly faster and entered their prepupal phase at 16 DAH, resulting in a lower uptake of microplastics^20^.

### Quantification of the microplastic excretion by BSF larvae

**Figure 4:**
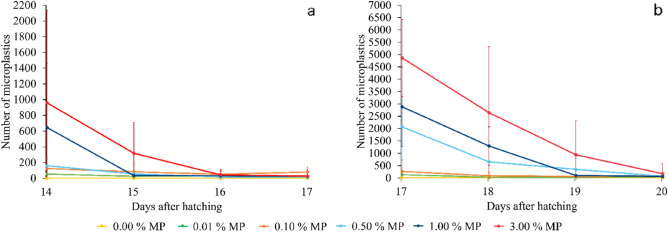
Retention of microplastics (MP) by BSF larvae reared on an artificial food waste stream containing different microplastic contents (*w*_MP_ = 0.00, 0.01, 0.10, 0.50, 1.00, 3.00%) of fluorescent blue-labelled polyethylene microplastics. (**a**) Represents the retention of microplastics starting at 14 DAH and (**b**) depicts the retention of microplastics starting at 17 DAH, both by showing the number of retained microplastics in the gut of the BSF larvae. The results shown are the mean values (*n* = 9), while the error bars depict standard deviations.

After the uptake, microplastic excretion was also assessed by quantifying the retained microplastics. For this experiment, the larvae were starved for three consecutive days at two different time points (at 14 and 17 DAH), by transferring them to a new and clean rearing container. During that period, the larvae were not provided with any substrate, while the retention of the microplastics was monitored by counting the remaining particles in their gut (Fig. 4). As with the uptake, excretion showed a similar pattern for all microplastic contents. As a result of the starvation process, the number of particles retained in the gut strongly decreased, but never went completely to zero. The final obtained number of microplastics in the BSF guts were 173 ± 402 (*w*_MP_ = 3.00%), 60 ± 149 (*w*_MP_ = 1.00%), 66 ± 126 (*w*_MP_ = 0.50%), 83 ± 109 (*w*_MP_ = 0.10%), and 54 ± 91 (*w*_MP_ = 0.01%), corresponding to 59%, 69%, 97%, 98%, and 96% excretion, respectively. The final numbers of microplastics retained in the gut also appear to be dependent on the initial microplastic content in the larval gut before the start of the starvation procedure.

### Bioaccumulation of microplastics

Despite the uptake of microplastics from the substrate, there was no evidence of accumulation in the gut of the larvae. Moreover, bioaccumulation factors (*BAF*) were all below one (Table 1), except for the lowest initial microplastic content (*BAF* = 1.07 ± 1.55 for *w*_MP_ = 0.01% at 17 DAH). As a result, it can be stated that during the rearing phase, BSF larvae not only ingested but also excreted those particles. Furthermore, the higher *BAF* values found for the lowest initial microplastic content also had a greater standard deviation. Indeed, small variations in the amounts of microplastics in the gut may lead to relatively high *BAF* values, as the initial microplastic content was low. As a consequence, these results should be treated with caution and firm conclusions are still difficult to draw.

**Table 1:** Bioaccumulation factors (*BAF*) of black soldier fly larvae reared on an artificial food waste stream containing different microplastic contents.

Microplastic content [%]	Bioaccumulation factor			
	14 DAH	17 DAH*	17 DAH	20 DAH*
0.00	0.00 ± 0.00^A/1^	0.00 ± 0.00^A/1^	0.00 ± 0.00^A/1^	0.00 ± 0.00^A/1^
0.01	0.61 ± 0.49^A/2^	0.25 ± 0.28^A/2^	1.07 ± 1.55^A/2^	0.54 ± 0.88^A/2^
0.10	0.13 ± 0.12^A/1^	0.13 ± 0.11^A/2^	0.22 ± 0.40^A/1,2^	0.09 ± 0.12^A/1,2^
0.50	0.04 ± 0.02^A/1^	0.01 ± 0.01^A/1^	0.33 ± 0.23^B/1,2^	0.01 ± 0.03^A/1^
1.00	0.06 ± 0.08^A/1^	0.01 ± 0.01^A/1^	0.26 ± 0.17^B/1,2^	0.01 ± 0.01^A/1^
3.00	0.04 ± 0.04^A/1^	0.00 ± 0.00^B,C/1^	0.12 ± 0.04^C/1^	0.01 ± 0.01^A,B/1^

*After three days of starvation.

The letters (A,B,C) depict a significant difference between different columns within the same row, while the numbers (1,2) represent a significant difference between different rows within the same column. The results shown are the mean values (*n* = 9), together with their standard deviations.

The *BAF*s of the starved larvae (Table 1) were generally halved after three days of starvation for the lower initial microplastic contents (*w*_MP_ = 0.01% and 0.10%), and became almost zero for the higher initial contents (*w*_MP_ = 0.50%, 1.00%, and 3.00%). It can be concluded that the larvae excreted microplastics and consequently lowering *BAF* values. On the other hand, although BSF larvae excreted the microplastics, there were still some particles present in their gut even after three days of starvation. As a result, it can be hypothesised that the application of BSF larvae containing microplastics as an ingredient in compound feed could potentially result in chemical safety issues, depending on the number of particles present in the larvae, and on the types of plastics and their accompanying additives.

Further research could be executed to optimise the excretion process, for instance, by transferring the larvae to a plastic-free substrate at the end of the rearing cycle. In addition, plastic materials could contain additives like plasticisers, antioxidants, flame retardants, etc., which could be released and accumulated in the larval gut. A chemical safety assessment should thus be performed to reveal whether the larvae accumulate, metabolise, and/or excrete such additives to get an accurate view on possible hazards.

### Effect of microplastics on the growth of the BSF larvae

**Figure 5:**
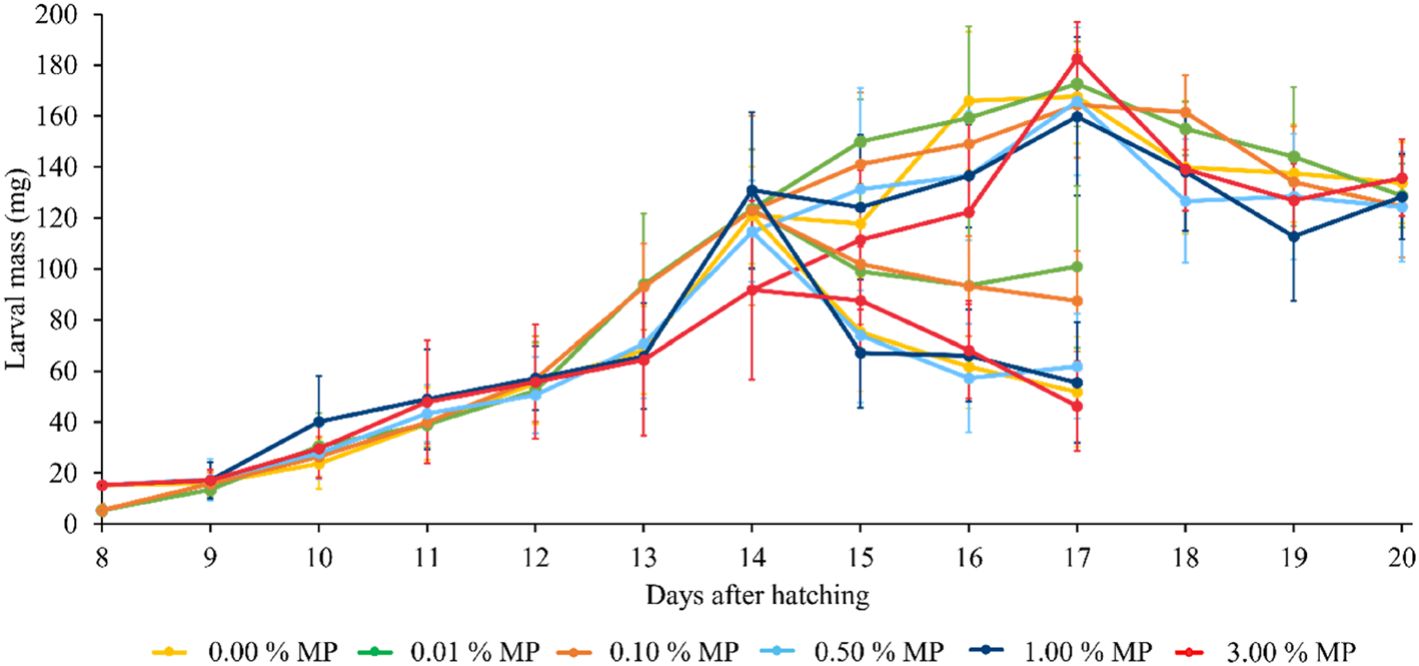
Growth curves of BSF larvae reared on an artificial food waste containing different microplastic contents (*w*_MP_ = 0.00, 0.01, 0.10, 0.50, 1.00, 3.00%) of fluorescent blue-labelled polyethylene microplastics (MP). At 14 and 17 DAH starvation started. The results shown are the mean values (*n* = 9), while the error bars correspond with their standard deviations.

In addition to the dynamics of the microplastic ingestion and excretion, the larval growth was simultaneously measured (Fig. 5). Based on the daily obtained larval mass recorded (*n* = 9), larvae were not affected in terms of growth by the presence of polyethylene microplastics. These results are similar to those obtained in other studies investigating the growth performance of BSF larvae reared on substrates containing other microplastics (e.g. polyvinyl chloride, polyethylene or polystyrene)^13,21^. By combining results reported in literature with our results, a hypothesis can be given as to why larval growth was not affected. Since microplastics were present in the larval gut and these particles did not affect BSF larvae in terms of growth, it seems likely that the exposure time may have been too short to express such effects, but this cannot be investigated due to the short larval lifespan. Instead, expression of some effects might take longer to occur, like for example during the pupal phase or in the next generation. Therefore, a study examining the entire BSF cycle for several generations is recommended. On the other hand, the larvae might also genetically adapt to thrive on substrates containing microplastics, which could also be investigated. Further, it can also be hypothesised that the microplastics act as an inert material, leaving the larvae unaffected. To investigate whether microplastics act as an inert material, or the exposure time is too short to have an effect on larval growth, immunological studies could be performed in future research to establish whether microplastics can induce an immune response in BSF larvae.

### Head morphology and mouth opening of BSF larvae

**Figure 6:**
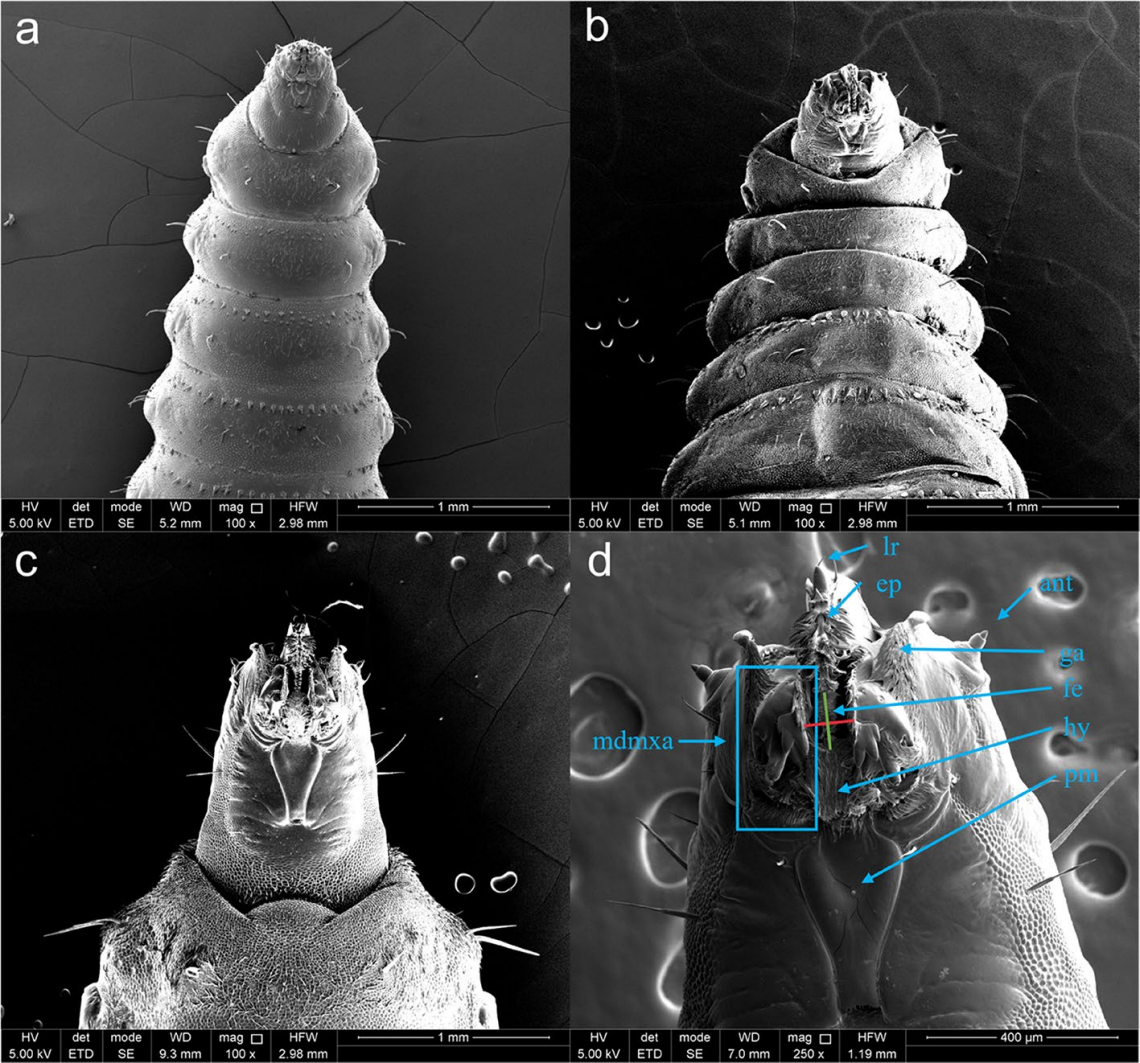
Ventral view of an (**a**) 5 DAH, (**b**) 8 DAH, and (**c**) 17 DAH old BSF larva using the same scanning electron microscopical conditions (magnification: 100 x). (**d**) shows a zoom of the head/mouth of a 15 DAH old BSF larva (magnification: 250 x). The red and green lines represent the mouth opening in transverse and median planes, respectively. lr = labrum, ep = epipharynx, ant = antenna, ga = galea, fe = food entrance, hy = hypopharynx, pm = prementum and mdmxa = mandibular-maxillary apparatus.

After microplastic uptake and excretion, the head morphology and larval mouth opening were investigated. Figure 6 depicts the ventral view of three BSF larvae at different ages (a: 5, b: 8, and c: 17 DAH). These images show the growth of the larvae and their mouth opening as they develop. Figure 6d displays a close-up image of a larva at 15 DAH, on which the different mouthparts are indicated. The antennae are short protuberances fulfilling sensory functions, while the labrum is a keel-shaped structure protecting the mandibular-maxillary apparatus, which helps larvae to move through the substrate. The epipharynx, which is connected to the labrum, contains a plethora of downwards-directed spines and continues into the mouth opening or food entrance, while the hypopharynx extends the mouth floor anteriorly, which is located below the food entrance zone. The prementum is, in turn, linked to the hypopharynx, fulfilling the function to remove heavier/bigger particles by its movements. As a result, it might also push away bigger plastic particles. The mandibular-maxillary apparatus allows the larvae to take up the semi-liquid substrate. It merges the mandibula and maxilla, which can be recognised by the three-toothed hook and a swollen membrane-like cylinder covered with a rasp, which grinds the food by a vertical movement. On the outside of the mandibular-maxillary apparatus, the galea are located, which contain different fans of spines depending on the larval stage^22,23^.

**Figure 7:**
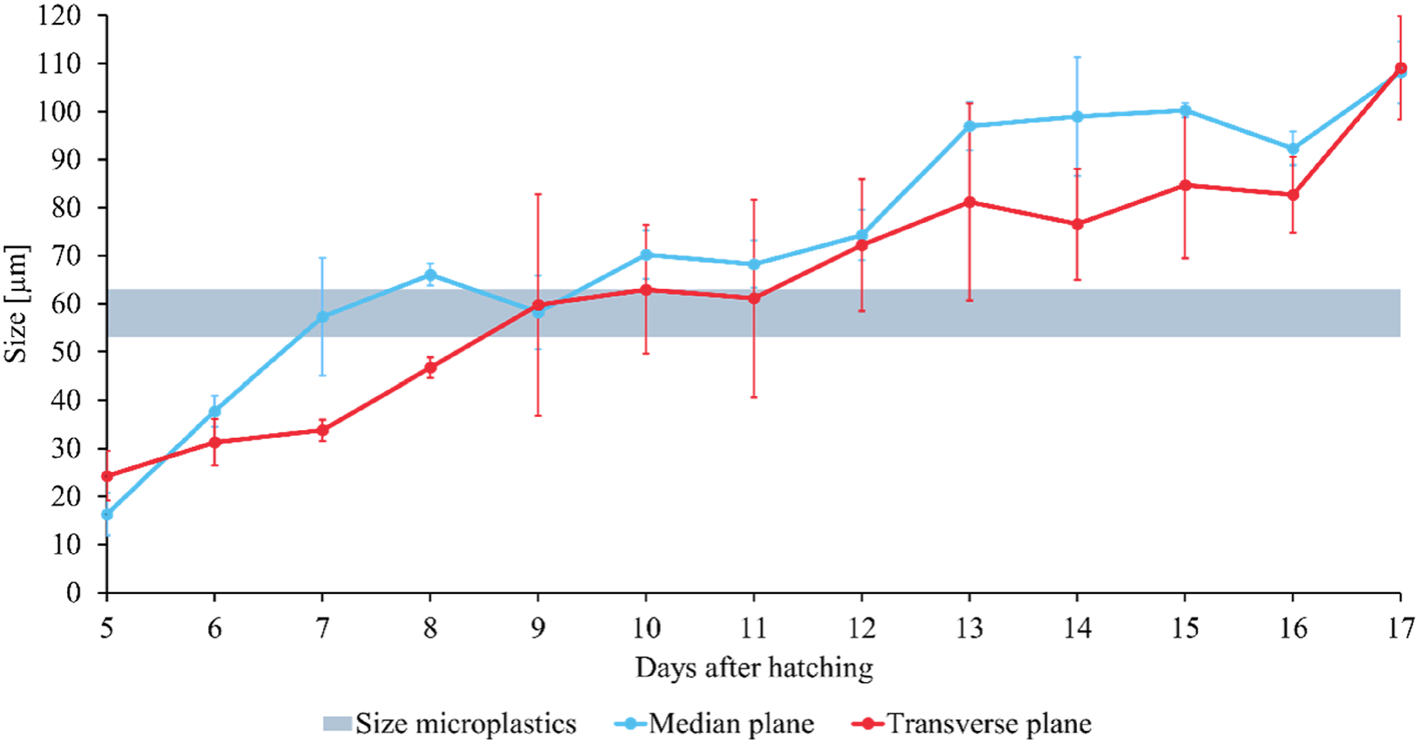
Larval mouth opening (median and transverse plane) as a function of their age. The size of the microplastics (53–63 µm) is depicted by the grey bar. The results provided are the means (*n* = 3), while the error bars display the standard deviations.

During the uptake assessment, it was established that the larvae start taking up microplastics at 10 DAH. To find out why the larvae started ingesting particles from that day, the larval mouth opening was measured. Figure 7 shows both the transverse and median plane of the larval mouth opening as a function of age and the size of the microplastics. The size of the mouth opening increased with age, starting from approximately 20 µm at 5 DAH and going to 110 µm at 17 DAH. From 10 DAH, the mouth opening (≈ 65 µm) outpaced the plastic particles (median size ≈ 61.5 µm) present in the substrate, allowing the larvae to ingest the microplastics. These results will allow researchers to predict whether microplastics, having a specific size, could be ingested by BSF larvae. Nevertheless, further confirmation is required for other plastic types, particle sizes and irregularly shaped microplastics. Additionally, even though the larvae grind their food before ingestion, it seems, based on the results obtained herein, that they were not able to grind the polyethylene microplastics, which would have led to an earlier uptake. This evidence also leads to the tentative conclusion that the larvae seem to only be able to ingest particles of their feeding substrate that are smaller than their mouth opening size. Therefore, the uptake of the microplastics strongly depends on the particle size and content in the substrate and the larval age or size. This is generally also the case for other organisms (e.g. *Chironomus riparius, Daphnia magna, Physella acuta, Crassostrea gigas,* zooplankton), where uptake was also dependent on the initial concentration, particle size and age of the organisms, but it had not yet been shown for insects frequently used as food and feed^14,16,17,24,25^. In turn, this could have implications for substrate processing as well. Reducing the particle size in the substrate could facilitate improved uptake by the larvae, which requires thorough investigations.

## Conclusions

Our findings in this study confirmed the ingestion and excretion of microplastics by BSF larvae by means of different microscopical techniques. The dynamics of both ingestion and excretion were dependent on the initial microplastic content in the substrate, but bioaccumulation did not occur. The vast majority of microplastics in the gut could be removed by introducing a fasting period just before harvesting the larvae. If complete elimination of microplastics is required, however, the starvation procedure needs to be further optimised. Further, it was observed that ingestion of the microplastics also depends on the size of the larval mouth opening, which must be larger than the available microplastics, confirming the proposed hypothesis. In conclusion, hardly digestible nursing feed might be improved by reducing the particle size, allowing the larvae to ingest it more easily, but future research on this topic is still recommended.

## Materials and methods

### Rearing black soldier fly larvae

BSF larvae were obtained from the research colony of RADIUS (Thomas More University of Applied Sciences, Belgium) and reared according to Broeckx et al.^26^. Briefly, eggs were collected, and approximately 1.0 g was hatched on 200 g chicken feed (AVEVE, Belgium) having a moisture content of 55%. After five days, another 600 g of chicken feed was added and, eight days after hatching (DAH), 150 larvae were counted and transferred into a round glass container (∅ = 7.5 cm, H = 10 cm; 3.3 larvae/cm^2^) having a lid with an opening of approximately 4 cm in diameter for ventilation. Following the transfer of the larvae, they were provided with their respective wet substrate (100 mg/larva/day) once at the beginning of the experiment. The removal of the larvae during the experiment was considered when calculating the amount of feed given to the larvae at the beginning of the experiment. Further, the substrate was an artificial food waste prepared according to Lievens et al.^9^ and subsequently spiked with different microplastic contents (mass fraction of microplastics (MP) or *w*_MP_ = 0.00, 0.01, 0.10, 0.50, 1.00, and 3.00%) before providing it to the larvae. The microplastics were fluorescent blue-labelled polyethylene microplastics (Cospheric LLC, USA) having a particle size ranging from 53 to 63 µm (*ρ*_MP_ = 1.13 g/cm^3^). At 14 and 17 DAH, 20 larvae were collected, washed with tap water, and starved for three consecutive days to investigate the excretion ability of the BSF larvae. Excretion was determined by quantifying the retained particles during starvation. Further, all rearing experiments were executed in triplicate for 13 days, which included 10 days of rearing and 3 days of starvation, while the temperature and relative humidity were respectively kept at 27 °C and 60%. Due to practical reasons, the rearing experiment was divided into two groups of treatments, which were executed sequentially (i.e. *w*_MP_ = 0.00, 0.01 and 0.10%, and *w*_MP_ = 0.00, 0.50, 1.00 and 3.00%). As a result, two different cohorts of larvae were used.

### Sampling procedure of the BSF larval gut

During the rearing and starvation phase, three larvae from each container were sampled and washed using tap water each day. Afterwards, the larvae were weighed, anaesthetised on ice, and their guts were immediately isolated in a phosphate-buffered saline (PBS) solution according to Bonelli et al.^27^ using a stereo microscope (Leica Microsystems, EZ4W, Germany). The obtained guts were then placed on a microscopical object slide, covered with PBS buffer and a coverslip to prevent dehydration preceding the visualisation and quantification of the microplastics in the lumen of the gut.

### Microplastic characterisation, visualisation and quantification

Microplastics were characterised by their shape, median particle size, and particle size distribution using a fluorescence microscope (Leica Microsystems, DMLS, Germany and Zeiss, Axioplan, Germany), an FEI Nova NanoSem 450 FEG scanning electron microscope (The Netherlands), and a Malvern Mastersizer 3000 (UK), according to Lievens et al. (2022 and 2023)^13,28^. Briefly, for the fluorescent microscopical imaging, the microplastics were placed on a microscopical object slide covered by a coverslip. For the SEM analysis, the microplastics were placed on an aluminium stub and coated with a 5 nm-thick platinum-palladium layer using a high vacuum (turbomolecular pumped) coater (Q150T, Quorum, UK). The SEM images were then produced at 20 kV acceleration voltage with an ETD (Everhart–Thornley) detector, while all other parameters like magnification, working distance (WD) and horizontal field width (HFW) were displayed on the respective image. Lastly, the microplastics were suspended in continuously stirred water (3100 rpm) until an obscuration of approximately 5% was reached, prior to measuring the particle size distribution in triplicate^13,28^.

Prior to the quantification of the microplastics in the larval guts, the particles were first visualised to verify their presence in the lumen of the gut. This was done by analysing the gut using both a light and a fluorescence microscope (Leica Microsystems, DMLS, Germany and Zeiss, Axioplan, Germany, respectively) equipped with Olympus SC30 (Japan) and MoticamPro 252A cameras (Motic, Hong Kong), respectively. Finally, both images were overlaid by using Adobe Photoshop 24.0.1 (USA). The fluorescent microscope had a halogen lamp with a 96 HE BFP filter set (VWR, USA), and the excitation and emission wavelengths used were 380 nm and 439 nm, respectively. With the fluorescence microscope, it was possible to confirm that the observed particles were the spiked fluorescent microplastics, as other particles or gut parts do not emit fluorescent light at that wavelength. After visualisation, microplastics could be quantified by manually counting all particles using both microscopes. In addition to the quantification of microplastics in the larval guts, the bioaccumulation factor (*BAF*) was calculated. *BAF* describes the relative concentration of, in this case, microplastics in the larval body against the concentration in their food^29^. *BAF* was calculated (Eq. (1)), according to Walker (1990)^29^ to assess the potential accumulation of the microplastics in BSF larvae.

1 $$BAF = \frac{{w_{{\text{MP in larvae}}} }}{{w_{{\text{MP in substrate}}} }} = \frac{{N_{{\text{MP }}} \times { }100{ } \times { }4{ } \times { } \times { }\overline{r}_{MP}^{3} { } \times { }\rho_{MP} }}{{3{ } \times { }\overline{m}_{{{\text{BSFL}}}} { } \times { }w_{{\text{MP in substrate}}} }}$$

where: *w*_MP in larvae_ = final microplastic mass fraction in BSF larvae [g/100 g], *w*_MP in substrate_ = initial microplastic mass fraction in substrate ingested [g/100 g], *N*_MP_ = number of microplastics, $\overline{r }$_MP_ = median radius of the microplastics [m], *ρ*_MP_ = microplastic density [g/m^3^], ${\overline{m} }_{\mathrm{BSFL}}$ = mean BSF larval mass (wet) [g] (*n* = 9).

### Study of the head morphology and measurement of the mouth opening

In addition to the quantification of microplastics in the lumen of the gut, the larval mouth openings were measured throughout the rearing process to establish a correlation with the ingestion of microplastics. The larval head morphology and the transverse and median plane dimensions of the mouth opening were determined in triplicate by means of SEM using a slightly modified procedure compared to the method described for the characterisation of the microplastics: three larvae of the same age were placed on an aluminium stub, degassed using an Edwards degassing unit (UK), and coated with a platinum-palladium layer of approximately 5 nm using a high vacuum coater (Q150T, Quorum, UK). SEM images of these larvae were made at 5 kV acceleration voltage using an FEI Nova NanoSem 450 FEG (The Netherlands) equipped with an ETD detector and operated under high vacuum of 10^–6^ mbar. The other settings, i.e., magnification, working distance (WD) and horizontal field width (HFW), are shown on the respective images. SEM images from 5 to 17 DAH larvae were acquired, and the mean mouth opening size was determined daily using three different fresh larvae.

### Statistical analyses

To determine statistical differences of the microplastic ingestion and excretion, the results were statistically assessed using JMP Pro 16.0.0 (SAS), with a 5% significance level. A one-way ANOVA with a Tukey HSD post hoc test in case of equal variances was performed. If the variances were not equal (determined by an O’Brien test), the Welch’s ANOVA was executed. Lastly, a non-parametric Van der Waerden test with Steel–Dwass All Pairs as a post hoc test was performed if the data were not normally distributed. The latter was evaluated by the Shapiro–Wilk test.

## Data Availability

The datasets generated during the current study are available from the corresponding author on reasonable request.
